# Development of Starch-Based Antifungal Coatings by Incorporation of Natamycin/Methyl-*β*-Cyclodextrin Inclusion Complex for Postharvest Treatments on Cherry Tomato against *Botrytis cinerea*

**DOI:** 10.3390/molecules24213962

**Published:** 2019-11-01

**Authors:** Yuexi Yang, Chen Huan, Xianrui Liang, Sheng Fang, Jian Wang, Jie Chen

**Affiliations:** 1 School of Food Science and Biotechnology, Zhejiang Gongshang University, Hangzhou 310018, China; yyx_526@126.com (Y.Y.); huanchen@zjgsu.edu.cn (C.H.); fszjgsu@163.com (S.F.); kaiser712h@sina.com (J.W.); 2 College of Pharmaceutical Sciences, Zhejiang University of Technology, Hangzhou 310014, China; liangxrvicky@zjut.edu.cn

**Keywords:** starch-based antifungal coatings, natamycin/methyl-*β*-cyclodextrin inclusion complex, postharvest treatments

## Abstract

The application of natamycin as a natural fungicide in edible coatings is challenging because of its low aqueous solubility. In this study, the natamycin/methyl-*β*-cyclodextrin (N/ME-*β*-CD) inclusion complex was fabricated and incorporated into waxy corn starch-based coatings for postharvest treatments. The phase solubility of natamycin in the presence of ME-*β*-CD at 293.2 K, 303.2 K, and 313.2 K is determined and used to calculate the process thermodynamic parameters. The N/ME-*β*-CD inclusion complex was confirmed and characterized by FTIR and 1H NMR spectroscopy. The results indicated that the inclusion complex was formed and the hydrophobic part (C16-C26) of natamycin might be partially inserted into the cavity of ME-*β*-CD form the wide rim. The effects of N/ME-*β*-CD incorporated starch-based coatings (N/ME-*β*-CD S coatings) on postharvest treatments of cherry tomatoes were evaluated in vivo. The N/ME-*β*-CD S coatings could reduce weight loss, delay fruit ripening, and inhibit fruit decay caused by *Botrytis cinerea* in tomato fruit during storage.

## 1. Introduction

In recent years, edible coatings and films based on biodegradable starch have gained great attention because of their highly efficient and low cost [1]. To extend the application of pure starch coatings, many functional properties are imported by the addition of various substances [2]. It has been demonstrated that a starch surface coating with good barrier properties could delay ethylene production and consequently delay the ripening of fruit during storage [3,4]. However, pathogenic fungi infect fresh fruit and cause enormous fruit losses during storage [5,6]. The incorporation of fungicide in starch coatings is an effective method to control fungi development in fruit [7]. It also provides a way to maintain high concentrations of the fungicide only on the surface of foods.

Many natural compounds such as natamycin and some essential oils have been demonstrated with good antifungal capabilities and applied in postharvest treatments for fruits [8]. However, the inherent hydrophobicity of these compounds makes it difficult to form a homogenous coating in real applications [9]. The non-homogeneous distribution of these hydrophobic additives will lead to less antifungal efficacy in some parts and also a higher residual amount in other sites. To solve the problem, a number of efficient strategies to incorporate hydrophobic additives into edible coatings and films have been developed such as nanoparticles, emulsion and inclusion complexes [9,10,11,12,13,14,15,16]. Many studies [13,14,15,16] showed that the molecular inclusion complexes of hydroprobic additives with cyclodextrins, particularly *β*-cyclodextrin (*β*-CD) derivatives, could enhance their efficacy and facilitate applications in edible coatings. Sun et al. examined the incorporation of curcumin/*β*-cyclodextrin emulsion inclusions into gelatin-based coatings and their impact on the quality of grass carp fillets (GCFs) during storage [16]. Different natural antimicrobials such as citral and trans-cinnamaldehyde [15], thymol [17], and essential oils [18] were also reported to be encapsulated in *β*-CD and used for food preservation. The lipophilic cavity of *β*-CD can provide appropriate size and non-polar environment, which makes it a very suitable carrier used to fabricate functional coatings. However, the low aqueous solubility of *β*-CD makes it difficult to be directly dissolved in the film casting procedure or to incorporate a high content of additives into coating blends [19,20]. It has been known that methylated *β*-CD (ME-*β*-CD) has an aqueous solubility up to 50-fold higher than that of *β*-CD. In addition, methylation of the external OH groups at positions 2, 3, and 6 make the inner surface of *β*-CD less sterically hindered and more hydrophobic. The chemical modification consequently improves the encapsulation efficiency of hydrophobic cargoes [21]. Until now, the types of *β*-CD derivatives used as encapsulating carriers for coating and film materials are limited.

Natamycin, a natural fungicide produced by *Streptomyces natalensis*, plays an important role in preventing yeast and fungal contamination in the food industry. It is approved worldwide as a safe food additive. Natamycin was also successfully applied in postharvest treatment against different fungi, including *Aspergillus japonicus* and *Gilbertella persicaria* for blackberry [22], *Botrytis cinerea* and *Penicillium* expansum for grape berries and jujube fruit [23]. However, the low solubility of natamycin in water leads to a non-uniform distribution on the coated surface and reduces its effectiveness against fungal, thus, varies natamycin inclusion comlexes have been developed for different applications [24,25,26,27]. An efficient delivery system of natamycin will facilitate its usage in postharvest treatments.

In this study, the inclusion complex natamycin/ME-*β*-CD (N/ME-*β*-CD) was characterized and used as an antifungal additive in corn starch coatings for the postharvest treatment of cherry tomato. The phase solubility of natamycin in the presence of ME-*β*-CD was determined, and thermodynamic parameters were obtained. The N/ME-*β*-CD inclusion complex was characterized by FTIR and NMR spectroscopy. The N/ME-*β*-CD inclusion complex was incorporated into waxy corn starch and applied as antifungal coatings (named N/ME-*β*-CD S coatings) in postharvest treatment of cherry tomato. The impacts of N/ME-*β*-CD S coatings on the ripening of green cherry tomatoes were evaluated and their capacity for controlling postharvest cherry tomato rot caused by *Botrytis cinerea* were tested.

## 2. Results and Discussions

### 2.1. Phase Solubility Studies of N/ME-β-CD Inclusion Complex

**Figure 1:**
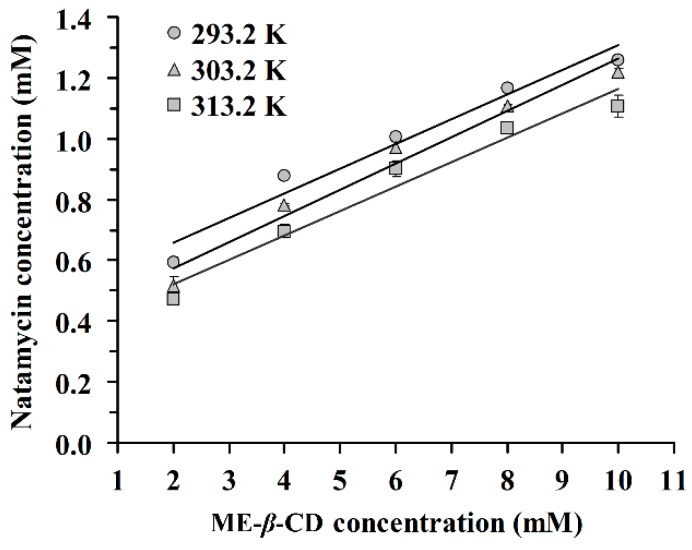
Phase solubility diagram of natamycin in the presence of Methyl-*β*-Cyclodextrin (ME-*β*-CD) at 293.2 K, 303.2 K, and 313.2 K.

The phase solubility of natamycin in the presence of ME-*β*-CD at 293.2 K, 303.2 K, and 313.2 K is shown in Figure 1. The results demonstrated that the concentration of natamycin in aqueous solution increased with increasing concentrations of ME-*β*-CD. According to the phase solubility profile [28], an A-type phase-solubility profile is observed for the N/ME-*β*-CD inclusion complex. This result indicates that the inclusion complex exhibits a 1:1 binding stoichiometry. The 1:1 drug/CD complex is the most common type found in reference [29] where one drug molecule forms a complex with one CD molecule.

On the other hand, a negative deviation from linear in the solubility curve of natamycin/ME-*β*-CD was observed, which indicated an A_N_-type phase solubility diagram. We hypothesized that aggregation behaviors may exist for natamycin or ME-*β*-CD in solution, especially at high concentrations. It is interesting to find that a dimer structure of natamycin ([2M-H]^-^ with mass 1329.6011) can be observed in the high-resolution mass spectrum of pure natamycin (Figure S1). It has been reported that amphotericin B, as an analog compound of natamycin, formed dimer due to pairwise association of the hydrophobic polyene chain [30]. Dimers are unable to form CD inclusion complexes [31]. Overall, the A_N_-type phase-solubility profile is difficult to interpret [28].

The inclusion complex stability constants (K_1:1_) were calculated from the slope and the intercept of the fitted straight line according to equation (1). The corresponding K_1:1_ values are summarized in Table 1. The K_1:1_ values increase with increasing temperature. The values of K_1:1_ is between 178 M^−1^ and 486 M^−1^, which are in agreement with the ranges (between 50 M^−1^ and 2000 M^−1^) described by other authors [21,32]. These values indicate that ME-*β*-CD forms a moderate or weak inclusion complex with natamycin in aqueous solution. Connors [33] reported 490 M^−1^ as an average K_1:1_ value for drug-*β*-CD complexes. In cases of very high K_1:1_ values (>5000), the inclusion complexes are very stable and the release of the drug molecule from the CD cavity is incomplete or obstructed. It has been reported that complexes with stability constants between 100 M^−1^ and 5000 M^−1^ are suitable for practical applications [33]. The apparent Gibbs free energy (Δ*G*_appearent_) of the inclusion complexation process is also listed in Table 1. The negative Δ*G* values demonstrated the spontaneity of the complexation reaction between natamycin and ME-*β*-CD. This result suggests that ME-*β*-CD with a hydrophobic cavity could offer a favourable environment for natamycin.

### 2.2. Characterizations and Interaction Mode of Natamycin/ME-β-CD Complex

**Figure 2:**
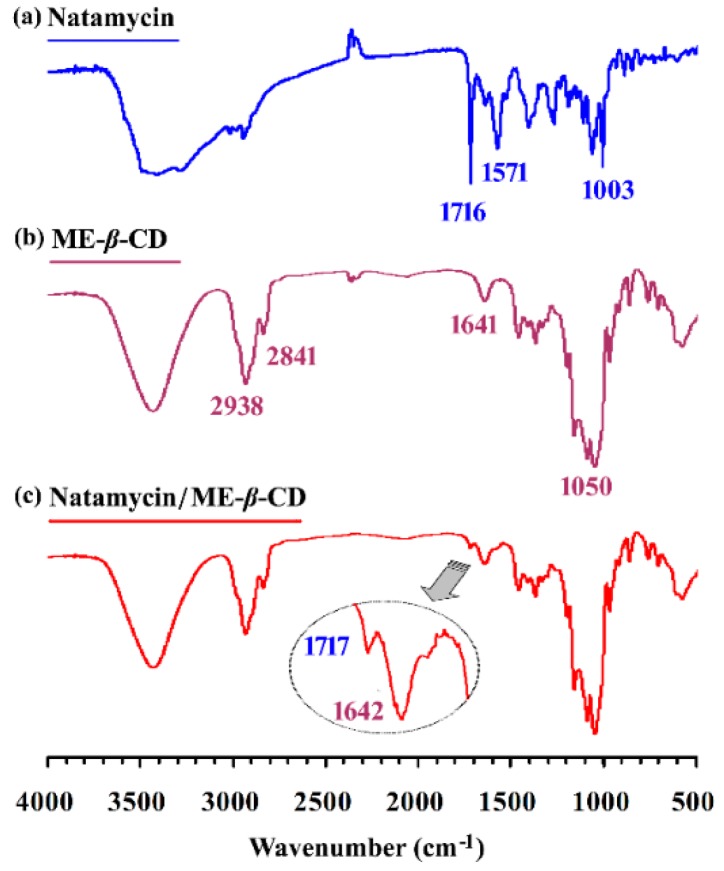
The FTIR spectrum of (a) natamycin, (b) Methyl-*β*-Cyclodextrin (ME-*β*-CD), and (c) natamycin/ME-*β*-CD inclusion complex.

The FTIR spectrum of natamycin, ME-*β*-CD, and the natamycin/ME-*β*-CD complex are shown in Figure 2. Many prominent peaks can be characterized for pure natamycin [34], such as the peaks at 1006 and 1267 cm^−1^ for cyclic ether, the peak at 1572 cm^−1^ for primary amine, and the peak at 1716 cm^−1^ for conjugated ester. The peaks at 2841 and 2938 cm^−1^ belong to the aliphatic C–H region of ME-*β*-CD. The bands in the range of approximately 1050 cm^−1^ can be associated with the stretching frequency of primary and secondary C–OH groups of ME-*β*-CD. There are no new peaks found in the spectrum of complexes other than those of natamycin and ME-*β*-CD. Compared with the spectrum of pure ME-*β*-CD, a new small peak at 1717 cm^−1^ can be found for the complex. As noted above, the conjugated ester absorption band (1716 cm^−1^) is the most informative one and appears for natamycin. This result indicates the successful inclusion of natamycin by ME-*β*-CD without chemical bond formation.

**Figure 3:**
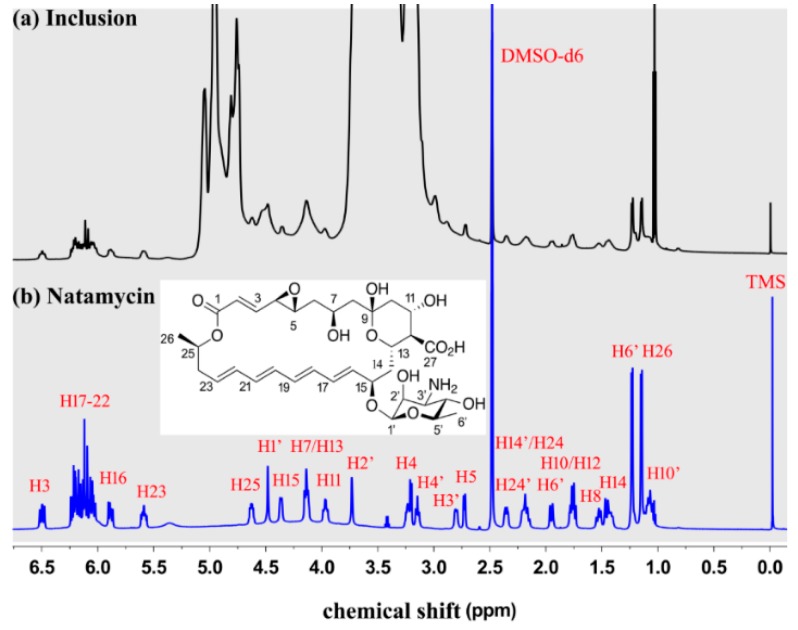
The 1H NMR spectrum of natamycin/ME-*β*-CD inclusion complex (**a**) and natamycin (**b**) in DMSO-d6.

The 1H NMR spectroscopy was used to characterize natamycin, ME-*β*-CD, and the natamycin/ME-*β*-CD inclusion complex [35]. Preliminary attempts showed that no sufficiently high concentration can be achieved to obtain an adequate 1H NMR signal of natamycin using D_2_O as a solvent due to its poor aqueous solubility, while natamycin/ME-*β*-CD inclusion complex was dissolved well both in D_2_O and DMSO-d6. Therefore, natamycin and natamycin/ME-*β*-CD inclusion complex was dissolved in DMSO-d6 for the 1H NMR studies. The 1H NMR spectrum of natamycin and the natamycin/ME-*β*-CD inclusion complex in DMSO-d6 is shown in Figure 3. The structure and peaks assigned to each H atom of natamycin can be seen. The assignments of each H are resolved with the help of the 1H-1H Correlated spectroscopy (COSY) experiment and compared with the available spectrum from references [36]. Although the signals are not very strong, the results again suggest that natamycin is successfully included in the complex.

The chemical shift variation (∆δ) of certain protons can be an indicator of its surrounding environment changes [37,38]. As seen from Table 2, the H14, H15, H16 atoms at the polyene macrolide part and the H1′ atom at the mycosamine part show higher absolute ∆δ values (>0.03 ppm) than other H atoms. It indicates that this part in the polyene macrolide structure is more influenced in the presence of ME-*β*-CD molecule [37]. We hypothesize that intermolecular hydrogen bonding’s are formed between the oxygen atom of C15–O–C1′ and the hydroxyl groups located at the rim of ME-*β*-CD. The C15–C16 part is more likely located near the rim of the cavity of CD ring. It is reasonable that the hydrophobic part (C16-C26) of natamycin is included in the hydrophobic cavity of CDs [24,35].

As ME-*β*-CD and natamycin/ME-*β*-CD inclusion complex was both dissolved well in D_2_O, D_2_O was selected as the solvent for the 1H NMR measurements. The molecule structure and peaks assigned to the ME-*β*-CD are shown in Figure S2. The chemical shifts of ME-*β*-CD in a free state and in natamycin/ME-*β*-CD inclusion complex are listed in Table 3. The results show that the surrounding electrons of H3 and H5 are more affected than the other H atoms of ME-*β*-CD (higher absolute ∆δ values). The H3 and H5 are the protons in the hydrophobic cavity of ME-*β*-CD. The upfield shifts observed for these two atoms can be seen as direct evidence for the formation of inclusion complex [39,40]. These results indicate that the natamycin was successfully inserted into the cavity of ME-*β*-CD.

When the absolute ∆δ value of H3 is larger than that of H5, partial inclusion of the guest inside the cavity of CDs occurs [41]. As a result, there is a partial inclusion of natamycin by ME-*β*-CD. Furthermore, the H3 is close to the wide rim of the hydrophobic cavity of CDs, while the H5 is close to the narrow side. These results suggest that the hydrophobic part (C16-C26) of natamycin is partially inserted into the cavity of ME-*β*-CD form the wide rim.

**Figure 4:**
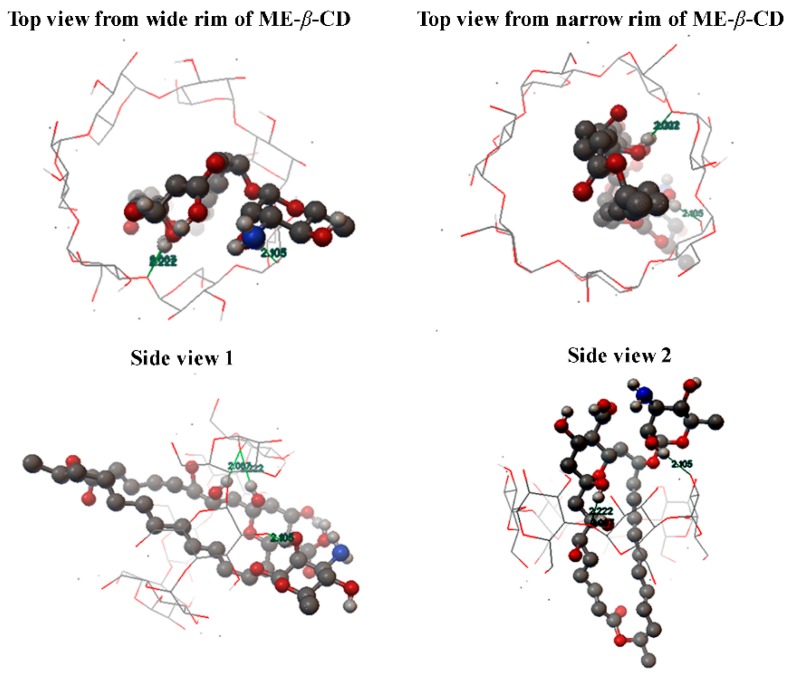
The proposed molecular inclusion mode of the natamycin/ME-*β*-CD inclusion complex.

Based on the above discussion, the molecular inclusion mode between natamycin and ME-*β*-CD is proposed and shown in Figure 4. The molecular docking method is showed in Method S1. However, detail structure conformation and interactions should be studied by molecular dynamics.

### 2.3. Impact of Coatings on the Weight Loss and Color Change of Cherry Tomato

**Figure 5:**
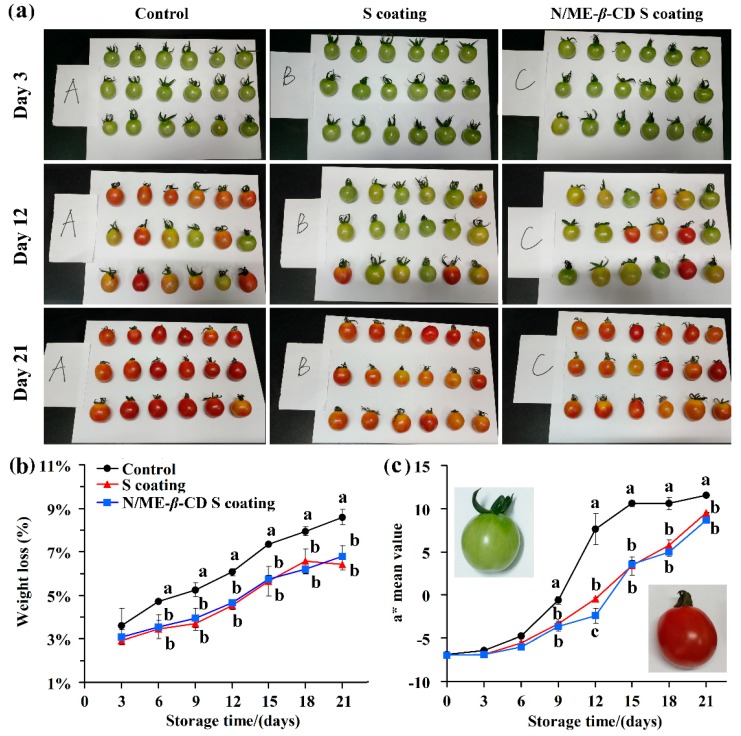
The selected appearance (**a**), weight loss (**b**), and color parameter a* value (**c**) of tomatoes from the control group, waxy corn starch-based coating (S coating) group, and natamycin/ME-*β*-CD incorporated waxy corn starch-based coating (N/ME-*β*-CD S coating) group at different storage times (days). The different lower-case letters at each time point indicate a significant difference at *p* < 0.05.

The weight loss and color change of cherry tomato with and without coatings were recorded during fruit ripening. As seen in Figure 5, all tomato fruits exhibited the same reduction profile. The control one (uncoated) showed a higher weight loss than the coated samples. It suggests that the starch-based coatings provide a protective layer and consequently reduce the water loss and rates of respiration. The result is comparable with the recent reports of starch-based edible coatings for fruit [3,42]. Nawab, Alam and Hasnain [4] revealed that the Mango kernel starch-based coating could effectively act as a water vapor protective layer on tomatoes and retard its ripening during twenty days storage. There is no significant difference in weight loss between the waxy corn starch-based coating (S coating) group and the natamycin/ME-*β*-CD incorporated waxy corn starch-based coating (N/ME-*β*-CD S coating) group (*p* > 0.05). This indicates that the natamycin/ME-*β*-CD inclusion complex can dissolve and distribute well in the starch-based coatings and will not affect the protective properties of the coatings. Naeem et al. [43] also pointed out that the addition of natural spice extracts in guar gum-based edible coatings did not influence its functionality on tomato.

The a* value range is negative for green to positive for red, which is a good indicator in determining the ripening stage of tomato fruit [44,45]. As can be seen in Figure 5a,c, the coated and uncoated fruit were predominantly red during storage (21 days) with an increasing tendency of a* values. However, the two kinds of starch-based coatings decrease the a* values of tomato fruit at the same storage time. It clearly demonstrates that the coatings delay the ripening process of tomato fruit and no significant differences (*p* > 0.05) were found for the S coating and the N/ME-*β*-CD S coating treated fruit. Again, the incorporation of natamycin/ME-*β*-CD into waxy corn starch-based coatings did not affect the functionality of coatings.

### 2.4. Effect of Coatings on the Growth of Botrytis cinerea In Vivo

**Figure 6:**
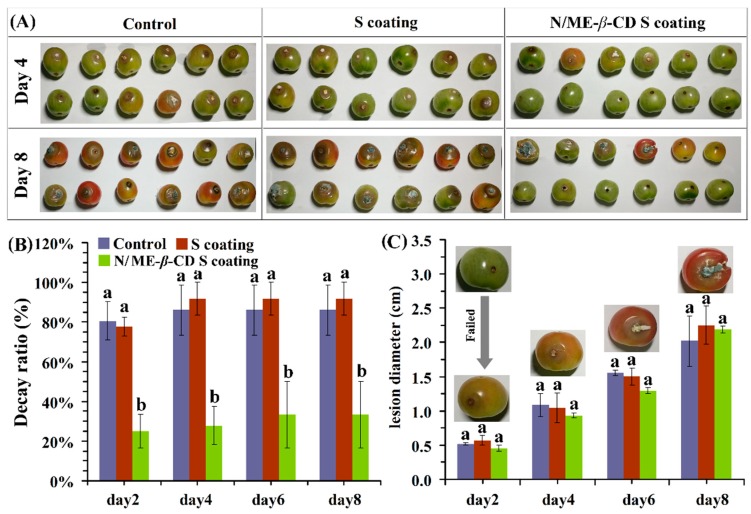
The selected appearance (**A**), decay ratio (**B**), and lesion diameter (**C**) of tomatoes from the control group, waxy corn starch-based coating (S coating) group, and natamycin/ME-*β*-CD incorporated waxy corn starch-based coating (N/ME-*β*-CD S coating) group after incubated with *Botrytis cinerea* at different storage times (days). The different lower-case letters at each time point indicate a significant difference at *p* < 0.05.

For the external validation, the effects of natamycin/ME-*β*-CD incorporated waxy corn starch-based coating (N/ME-*β*-CD S coating) on the postharvest grey mold of cherry tomatoes were studied and shown in Figure 6. In this study, tomatoes were inoculated with a given volume of a conidial suspension after coating. The decay ratios of control, waxy corn starch-based coating (S coating) treated and N/ME-*β*-CD S coating treated samples are about 86 ± 12%, 91 ± 8%, and 27 ± 9% after 4 days incubation, respectively. The S coating samples showed no significant differences with the control (*p* > 0.05). It shows that the treatment of N/ME-*β*-CD S coatings could significantly reduce the decay rate of postharvest cherry tomatoes and thus maintained the fresh-preservation effect. Similar results had been reported in grape that natamycin coating could better maintain fruit fresh and prolong storage period [46].

It can be seen from Figure 6C that the lesion diameter of tomato fruit in all groups increased with the prolongation of storage time, and the rising trend of the lesion diameter was similar for all three samples. However, previous research suggested that natamycin coating showed significantly higher antimicrobial effect against yeast and mold in strawberries [47]. Given the difference of the surface between tomatoes and strawberries, it is supposed that when natamycin is not in full contact with the fungi, the microorganism cannot be killed and leads to the growth of gray mold on tomato surface as observed. We estimate that the coating methods may play an important role in the antimicrobial performance of N/ME-*β*-CD S coating. On the other hand, in vivo antifungal performance of fungicide drugs may also be affected by complex environmental parameters, such as hydrophobicity of surface, pH, and nutrition of coated plants. In addition, the covered concentration of natamycin could also affect its antifungal properties. These issues need more studies. Overall, the results indicate that the N/ME-*β*-CD S coating can inhibit gray mold rot caused by *Botrytis cinerea* in tomato fruit during storage.

## 3. Materials and Methods

### 3.1. Materials

Natamycin was obtained from Zhejiang Sliver-Elephant Bio-engineering Co., Ltd. (Tiantai, China). The natamycin was checked by high-resolution mass spectrometry according to the method reported by Xu.X. et al. [48]. Methyl-*β*-cyclodextrin (2,6 di-O-methyl-*β*-cyclodextrin) were purchased from Sigma Aldrich (Shanghai, China). *Botrytis cinerea* was conserved by our laboratory. Waxy corn starch with 10.82% moisture was purchased from China Oil & Foodstuffs Corporation (Beijing, China). The viscosity-average molecular weight of the waxy corn starch is determined as 1.56 × 10^6^ Da. All other reagents used were of analytical grade. Distilled water was used to prepare solutions unless otherwise specified.

### 3.2. Phase Solubility Studies

Phase solubility studies were conducted following the method reported by Higuchi and Connors [28]. A standard curve of natamycin in methyl alcohol was prepared beforehand. Excess amounts of natamycin were added to 10 mL aqueous solution of ME-*β*-CD ranging in concentrations from 0 to 10 mM. To achieve equilibrium, the solutions were then ultrasonicated (Shenzhen JATO science technologies, Co., Ltd., Shenzhen, China) for 5 min and mixed at 200 r/min for 24 h at 293.2 K, 303.2 K, and 313.2 K. Then, the solutions were filtered with a 0.45 µm filter and the quantity of natamycin was measured at 303 nm using a UV spectrophotometer (UV-2600, Shimadzu, Japan).

A straight line was fitted to the phase solubility data and the natamycin/ME-*β*-CD 1:1 equilibrium constant was determined. According to the Higuchi-Connors equation (Equation (1)), the stability constant (K1:1) was calculated according to Equation (1).

(1) $$K_{1:1}=\frac{slope}{S_{0}\times (1-slope)}$$

where *S*_0_ is the connatural solubility of natamycin in the absence of natamycin/ME-*β*-CD, obtained as the y-intercept [49], and the slope is obtained by the linear regression of phase solubility values (mM) against CD concentrations (mM).

The change in the apparent Gibbs free energy (∆*G*_apparent_) was determined as a function of the *K* and the temperature *T* according to Equation (2).

(2) $$\Delta G_{\mathrm{apparent}}=-RT\times lnK$$

### 3.3. FTIR Spectrum

The clear solution of the natamycin/ME-*β*-CD inclusion complex was prepared above and freeze-dried using a vacuum freeze dryer (Beijing biocool Laboratory Instrument Co. Ltd., Beijing, China) to obtain a solid powder. The solid samples were stored in dark and dry for further characterization.

The fourier transform infrared spectroscopy (FTIR) measurements of natamycin, ME-*β*-CD, and natamycin/ME-*β*-CD inclusion complex were conducted using an FTIR Spectrometer (Nicolet iS5, Thermo Fisher, Waltham, MA, USA). The diffuse reflectance technique was utilized in the range from 4000 cm^−1^ to 400 cm^−1^. The samples were ground with spectroscopic grade potassium bromide (KBr) powder and then pressed into 1 mm pellets.

### 3.4. NMR Spectrum

The proton nuclear magnetic resonance (1H NMR) spectra of natamycin, ME-*β*-CD, and natamycin/ME-*β*-CD complex were collected at 308 K using a Bruker Avance III 600 MHz NMR Spectrometer (Billerica, MA, USA). Two solvent systems were used. The natamycin and the natamycin/ME-*β*-CD complex were dissolved in DMSO-d6 for the NMR characterization of the 1H shift of the natamycin. The ME-*β*-CD and the natamycin/ME-*β*-CD complex were dissolved in D_2_O for the NMR characterization of the 1H shift of the ME-*β*-CD. The chemical shifts were described in ppm referenced to solvent. The poetical assignments of the 1H spectra for natamycin were also achieved by using 2D (1H, 1H) NOESY employing mixing times 400 ms. The chemical shifts (δ) were calculated by using Mestrenova software [50].

### 3.5. Preparation of Starch-Based Coating Materials and Pretreatment of Cherry Tomato

The aqueous solution of the natamycin/ME-*β*-CD inclusion complex was prepared firstly (method is shown in Figure S3), which contained 200 mg/mL natamycin and 1.25 g/mL ME-*β*-CD. Another aqueous suspension contained 6%wt waxy corn starch and 3%wt glycerol was prepared secondly. The above solution and suspension were mixed together at 1:1 ratio and stirred at 400 r/min for 60 min, followed by degassing with sonication at 100 W for 10 min, thus yielding the natamycin/ME-*β*-CD incorporated starch-based coating (N/ME-*β*-CD S) material. Waxy corn starch-based coating (S coating) material was an aqueous suspension contained 3%wt waxy corn starch and 1.5%wt glycerol.

Cherry tomatoes (L. esculentum cv. No. 3 Zhengyinfen) at the mature green stage were harvested from a local farm in Haining (Zhejiang Province, China) and transported to university lab. After 2 h of removing the field heat, the tomato fruit without injuries were carefully selected. The selected fruit was firstly disinfected with 1% NaClO solution, then cleaned with distilled water, and finally dried by airing for further experiments.

### 3.6. In Vivo Effects of N/ME-β-CD S Coating on the Weight Loss and Color Change of Cherry Tomato

The selected green cherry tomatoes were divided into 3 groups and each group had 54 tomatoes (three replicates with 18 cherry tomatoes). The tomato fruits in different groups were dipped in different coating materials for 5 min and then dried at room temperature. Group 1: distilled water (control); Group2: waxy corn starch-based coating (S coating); Group 3: natamycin/ME-*β*-CD incorporated waxy corn starch-based coating (N/ME-*β*-CD S coating). The mass of treated cherry tomatoes were recorded during storage. The weight loss (%) at each time point was calculated according to Equation (3).

(3) $$weight\;loss\;(\%)=\frac{W_{1}-W_{t}}{W_{1}}\times 100$$

where *W*_1_ equal to the weight of cherry tomato before storage, *W*_t_ means the weight at storage time point.

The color parameter a* represents redness to greenness were recorded for the control and coated samples during storage by using a Reflectance Chroma MEter CR 210 (Minolta Co. Ltd., Osaka, Japan). The equipment was checked with white and black boards before every test. More than three measurements at different positions were performed for each sample, and the average value was taken.

### 3.7. In Vivo Effects of N/ME-β-CD S Coating on Incubated Cherry Tomato against Botrytis cinerea

The 1.0 × 10^4^ CFU/mL spore suspension of *Botrytis cinerea* was prepared and used to inoculate the selected green cherry tomato. The selected tomatoes were divided into 3 groups and each group had 36 tomatoes (three replicates with 12 cherry tomatoes). Tomatoes in different groups were treated with different coating materials (grouping and treatments were the same as shown in Section 3.6). Then, a wound at the equator of tomato with both 3 mm wideand deep was made using a sterile borer. A certain amount (20 μL) of *Botrytis cinerea* suspension was added to the wound. The cherry tomatoes were incubated in a plastic box at 22 °C with 85~90% RH. The decayed tomato fruits were recorded and the disease diameter on each tomato fruit was measured for 8 days storage with 2 days intervals.

### 3.8. Statistical Analysis

All analyses were carried out in triplicate. The values were described as the means ± standard deviations. The data were subjected to the analysis of variance (ANOVA) by Origin 9.0 (OriginLab Co. Ltd., MA, USA). The significant was defined at the 95% confidence level (*p* < 0.05).

## 4. Conclusions

The phase solubility of natamycin in the presence of ME-*β*-CD was determined and used to calculate the thermodynamic parameters. The FTIR suggested the successful inclusion of natamycin by ME-*β*-CD without chemical bond formation. The 1H NMR results indicated that the hydrophobic part (C16-C26) of natamycin might be partially inserted into the cavity of ME-*β*-CD form the wide rim. The in vivo test showed that the natamycin/ME-*β*-CD S coating could reduce weight loss, delay fruit ripening, and inhibit gray mold rot caused by *Botrytis cinerea* in tomato fruit during storage. In future studies, more types of fungi and fruit models should be selected to evaluate the possibility of natamycin/ME-*β*-CD incorporated starch coatings for antifungal applications.

## Figures and Tables

**Table 1:** Stability constant (K_1:1_) and apparent Gibbs free energy (Δ*G*_appearent_) of the natamycin/Methyl-*β*-Cyclodextrin inclusion complex at different temperatures.

Temperature (K)	K_1:1_ (M^−1^)	∆*G*_appearent_ (kJ/mol)
293.2	178.06	−12.63
303.2	234.06	−13.75
313.2	240.88	−14.28

**Table 2:** Variation of the chemical shifts (δ, ppm) of natamycin in a free state and in natamycin/ME-*β*-CD inclusion complex in DMSO-d6.

Substance	Protons	Free *δ* (ppm)	In Complex *δ* (ppm)	∆*δ* ^1^ (ppm)
Natamycin	H3	6.5229	6.5003	−0.0225
	H17-H22	6.1439	6.1164	−0.0275
	H16	5.9283	5.8921	−0.0361
	H23	5.6116	5.5928	−0.0188
	H25	4.6517	4.6265	−0.0252
	H1′	4.5043	4.4666	−0.0377
	H15	4.3952	4.3635	−0.0317
	H7/H13	4.1609	4.1430	−0.0178
	H11	3.9928	3.9776	−0.0151
	H2′	3.7546	Covered	/
	H4	3.2542	Covered	/
	H5′	3.2355	Covered	/
	H4′	3.1765	Covered	/
	H3′	2.8291	Covered	/
	H5	2.7423	2.7190	−0.0233
	H24′	2.3713	2.3583	−0.0130
	H14′/H24	2.2077	2.1832	−0.0245
	H6	1.9623	1.9424	−0.0199
	H10/H12	1.7909	1.7621	−0.0288
	H8	1.5496	1.5346	−0.0151
	H14	1.4922	1.4441	−0.0481
	H6′	1.2593	1.2381	−0.0212
	H26	1.1750	1.1545	−0.0205
	H10′	1.0550	1.0350	−0.0200

^1^ ∆δ equals to the δ of certain proton in complex minus δ value of the same proton in natamycin.

**Table 3:** Variation of the 1H chemical shifts of ME-*β*-CD in a free state and in natamycin/ME-*β*-CD inclusion complex in D_2_O.

Substance	Protons	Free *δ* (ppm)	In Complex *δ* (ppm)	∆*δ* ^1^ (ppm)
ME-*β*-CD	H1	5.2450	5.2446	0.0000
	H2	5.0392	5.0381	−0.0011
	H3	3.9698	3.9582	−0.0116
	H4	3.6177	3.6169	−0.0008
	H5	3.8903	3.8869	−0.0033
	H6	3.6816	3.6820	0.0004

^1^ ∆δ equals to the δ of certain proton in complex minus δ value of the same proton in natamycin.

## Supplementary Materials

The following are available online at https://www.mdpi.com/1420-3049/24/21/3962/s1, Figure S1: Mass spectrum of natamycin and its dimer, Figure S2: The 1H NMR assignment of ME-*β*-CD and its molecular structure in D_2_O, Figure S3: Schematic diagram of the formation of N/ME-*β*-CD complex, Method S1: Molecular docking.
