# Supplementary material for: Development of Starch-Based Antifungal Coatings by Incorporation of Natamycin/Methyl-β-Cyclodextrin Inclusion Complex for Postharvest Treatments on Cherry Tomato against Botrytis cinerea
Source: Molecules. 2019 Nov 1;24(21):3962. doi: 10.3390/molecules24213962 (PMC6864615; doi:10.3390/molecules24213962)
Supplement: Supplementary file 1 [file molecules-24-03962-s001.pdf]

Supplementary Materials

Development of starch-based antifungal coatings by incorporation of natamycin/methyl-*β*-cyclodextrin inclusion complex for postharvest treatments on cherry tomato against *Botrytis cinerea*

Yuexi Yang [1], Chen Huan [1], Xianrui Liang [2], Sheng Fang [1], Jian Wang [1] and Jie Chen [1,*]

[1] School of Food Science and Biotechnology, Zhejiang Gongshang University, Hangzhou 310018, China; yyx_526@126.com (Y.Y.); huanchen@zjgsu.edu.cn (C.H.); fszjgsu@163.com (S.F.); kaiser712h@sina.com (J.W.); chenjie@zjgsu.edu.cn (J.C.)

[2] College of Pharmaceutical Sciences, Zhejiang University of Technology, Hangzhou 310014, China; liangxrvicky@zjut.edu.cn (X.L.)

* Correspondence: chenjie@zjgsu.edu.cn; Tel.: +86-1351-681-2119 (J.C.)

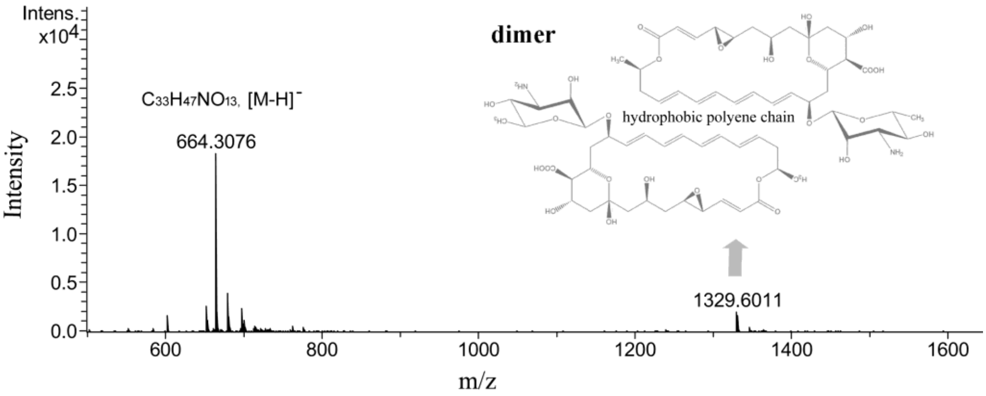

Figure S1. Mass spectrum of natamycin and its dimer.

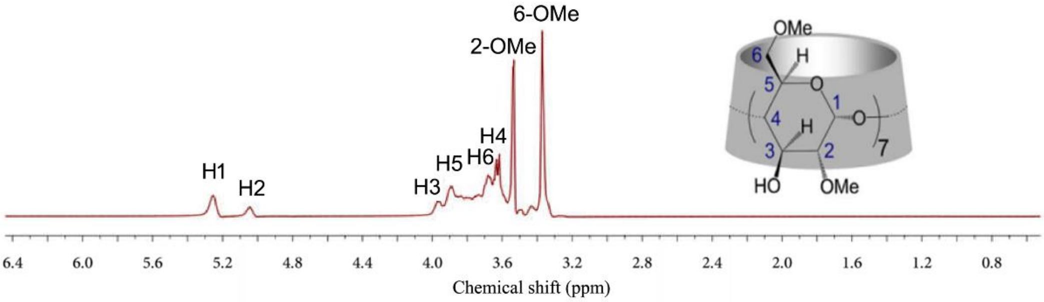

Figure S2. The 1H NMR assignment of ME-*β*-CD and its molecular structure in D_2O.

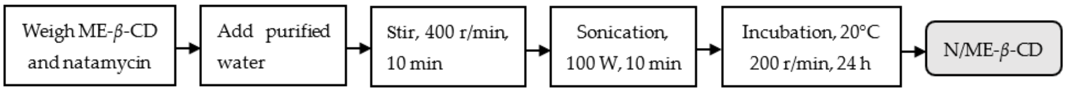

Figure S3. Schematic diagram of the formation of N/ME-β-CD complex.

Method S1: Molecular docking

The structures of β-CD and natamycin were got from the crystallographic parameters provided by the Structural Data Base System of the RCSB Protein Data Bank. The structure of ME-β-CD was modified using GaussView by adding 14 methyl groups in position 2 and 6 of the β-CD. Then the ME-β-CD molecule was optimized using PM3. Molecular docking study was carried out with the automated docking program (AutoDock 4.0.1) with Lamarckian genetic algorithm (LGA) [1,2]. AutoDock defines the conformational space implementing grids over all the possible search space. A grid of 100 Å by the side and 0.375 Å spacing between each point. The center of the ME-β-CD was set as the center of the box. The initial torsions and positions of natamycin were generated randomly. The ligand and receptor files were processed using the AutoDock Tools [1,2].

References：

1. Morris, G. M.; Goodsell, D. S.; Halliday, R.S.; Huey, R.; Hart, W. E.; Belew, R. K.; Olson, A. J. Automated Docking Using a Lamarckian Genetic Algorithm and and Empirical Binding Free Energy Function. *J. Comput. Chem.* **1998**, *19*, 1639–1662. doi: 10.1002/(SICI)1096-987X(19981115)19:14<1639::AID-JCC10>3.0.CO;2-B

2. Morris, G. M.; Huey, R.; Lindstrom, W.; Sanner, M. F.; Belew, R. K.; Goodsell, D. S.; Olson, A. J. Autodock4 and AutoDockTools4: automated docking with selective receptor flexiblity. *J. Comput. Chem.* **2009**, *16*, 2785–2791. doi: 10.1002/jcc.21256
